# Gauchos and ochos: a Wee1-Cdk tango regulating mitotic entry

**DOI:** 10.1186/1747-1028-5-12

**Published:** 2010-05-13

**Authors:** Greg H Enders

**Affiliations:** 1 Department of Medicine, Fox Chase Cancer Center, 333 Cottman, Philadelphia, PA 19111, USA; 2 Epigenetics and Progenitor Cell Keystone Program, Fox Chase Cancer Center, 333 Cottman, Philadelphia, PA 19111, USA

## Abstract

The kinase Wee1 has been recognized for a quarter century as a key inhibitor of Cyclin dependent kinase 1 (Cdk1) and mitotic entry in eukaryotes. Nonetheless, Wee1 regulation is not well understood and its large amino-terminal regulatory domain (NRD) has remained largely uncharted. Evidence has accumulated that cyclin B/Cdk1 complexes reciprocally inhibit Wee1 activity through NRD phosphorylation. Recent studies have identified the first functional NRD elements and suggested that vertebrate cyclin A/Cdk2 complexes also phosphorylate the NRD. A short NRD peptide, termed the Wee box, augments the activity of the Wee1 kinase domain. Cdk1/2-mediated phosphorylation of the Wee box (on T239) antagonizes kinase activity. A nearby region harbors a conserved RxL motif (RxL1) that promotes cyclin A/Cdk2 binding and T239 phosphorylation. Mutation of either T239 or RxL1 bolsters the ability of Wee1 to block mitotic entry, consistent with negative regulation of Wee1 through these sites. The region in human somatic Wee1 that encompasses RxL1 also binds Crm1, directing Wee1 export from the nucleus. These studies have illuminated important aspects of Wee1 regulation and defined a specific molecular pathway through which cyclin A/Cdk2 complexes foster mitotic entry. The complexity, speed, and importance of regulation of mitotic entry suggest that there is more to be learned.

## Introduction: Wee1 is a Cdk1 kinase

**Figure 1:**
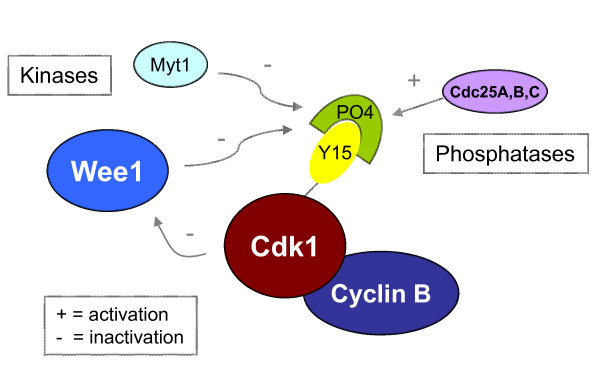
**Reciprocal regulation of Wee1 and Cdk1**. The feedback loop is a double-negative one, resulting in positive regulation of cyclin B/Cdk1 as the activity of this Cdk complex rises. Wee1 inhibits Cdk1 by phosphorylating it on tyrosine 15. Myt1 also performs this modification, though Wee1 appears to be dominant. Cdc25 phosphatases (A, B, C) remove the phosphate group. Cdk1 can also phosphorylate Wee1, inhibiting it.

Mitotic entry is the paradigmatic cell cycle transition and example of Cdk regulation. Yet, our understanding of this transition remains superficial. A long-term goal of research in this area is to design drugs that treat cancer by either blocking mitotic entry or driving cells into mitosis in the face of lethal DNA damage. Cyclin B/Cdk1 (Cdc2/Cdc28) complexes direct many of the events of mitosis. These events must be launched in swift, coordinated fashion but only after DNA synthesis is completed and DNA damage is repaired. To effect such control, cyclin B/Cdk1 activity is regulated through dynamic post-translational modifications. Wee1 is a universal Cdk1 inhibitor that phosphorylates a tyrosine residue (Y15) in the ATP binding site, thereby blocking Cdk1 activity (Fig 1). Research is unraveling an intricate dance executed by these two kinases and closely related Cdk complexes as they exert reciprocal regulation. This commentary focuses on recent advances in vertebrates, but leans also on elegant parallel studies in budding yeast of the interaction between Cdk1 (Cdc28) and the Wee1 homologue Swe1 [1]. In vertebrates, embryonic (Wee1B in most species, Wee1A in Xenopus) and somatic (Wee1A in most species, Wee1B or Wee2 in Xenopus) proteins are encoded by two distinct genes [2]. Functional differences between embryonic and somatic proteins are beginning to emerge (discussed below).

### Reciprocal regulation of Wee1 by Cdk1

Wee1 was discovered in yeast as the target of mutations that allow cells to divide at half their usual size [3]. Wee1 is regulated at multiple levels, including transcription [4], translation [5], and protein stability [6-10], but we focus here on recent progress made in understanding the effects of Cdk1/2 phosphorylation on Wee1 activity and localization. Wee1 becomes hyperphosphorylated during mitosis, accompanied by reduced activity [4,11]. Moreover, Wee1 was found to be directly inactivated by cyclin B/Cdk1 complexes *in vitro *[12], although this effect has remained controversial (see below) [4]. The net effect is a positive feedback loop (Fig 1) that could logically allow Cdk1 activity to increase rapidly, thereby facilitating prompt execution of the dramatic events of mitosis.

**Figure 2:**
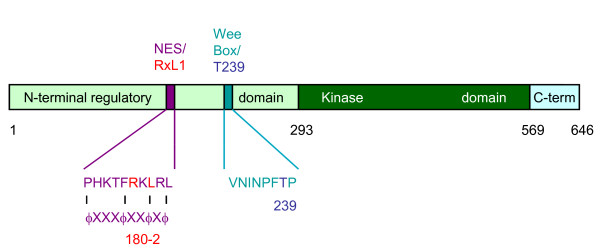
**Primary structure of vertebrate somatic Wee1 proteins**. The numbering is from human somatic Wee1. The NRD, kinase domain, and short carboxy-terminal domain are marked, with border amino acid residues numbered (below). RxL1 (residues 180-2) is embedded within the Crm1 binding site (175-184). The T239 Cdk phosphorylation site, an inhibitory modification, resides within the Wee box, a positive regulatory element.

Cyclin B/Cdk1 phosphorylation sites have recently been mapped in Swe1 and Xenopus embryonic Wee1 [1,13] (D. Kellogg, unpublished). The major sites each manifest the loose S/T-P Cdk consensus phosphorylation sequence. Two sites in Xenopus embryonic Wee1 were found to be conserved among vertebrate Wee1 species and functionally important for inhibiting Wee1 kinase activity. The site with stronger effect is T239, using the numbering system for human somatic Wee1 (Fig 2; T150 in Xenopus embryonic Wee1). This site becomes phosphorylated shortly before mitotic entry in cycling Xenopus egg extracts. A T239 mutant showed increased inhibition of mitotic entry in cyclin B-activated interphase extracts [13]. Xenopus somatic Wee1 is also phosphorylated at this site (T186 in that protein) [14]. Further studies revealed that an encompassing peptide termed the 'Wee box' (Fig 2) augments the activity of the kinase domain, *in cis *or *in trans *[14]. Interestingly, the Wee box is conserved in most eukaryotic Wee1 proteins but not Swe1. Cdk phosphorylation in Swe1 has thus far been shown to be activating rather than inhibiting during early phases of mitosis [1](D. Kellogg, unpublished). The absence of a Wee box offers a potential explanation for this different outcome in Swe1. Although mutation of T239 to alanine in Xenopus somatic Wee1 yielded a more potent blockade of mitosis, Cdk1-mediated phosphorylation *in vitro *did not inactivate Wee1 kinase activity [14]. Further studies revealed that T239 phosphorylation *in vivo *directs binding of the peptidyl-prolyl isomerase Pin1. Pin1 appears to inactivate Wee box function, although the detailed mechanism remains unknown [14]. In summary, these studies defined a pathway through which cyclin B/Cdk1 complexes negatively regulate Wee1.

### A role for cyclin A/Cdk2 complexes

Beyond this positive-feedback loop involving cyclin B/Cdk1 complexes, evidence has accumulated for a decade that the major Cdk complexes in the preceding S and G2 cell cycle phases--cyclin A (A2)/Cdk2 complexes--help pave the way for mitosis by reducing cyclin B/Cdk1 Y15 phosphorylation. Microinjection of cyclin A was observed to drive cultured human cells into mitosis and injection of cyclin A/Cdk2 inhibitors could block mitotic entry [15,16]. Induction of a dominant negative mutant of Cdk2 arrested cells in late S and G2 phase, associated with increased Cdk1 Y15 phosphorylation [17]. Similarly, cyclin A RNAi imposed a G2 arrest associated with increased Cdk1 Y15 phosphorylation [18]. Cdc25 phosphatases, the enzymes that reverse Cdk1 Y15 phosphorylation, showed reduced activity when cyclin A/Cdk2 complexes were inhibited [18]. Although augmented Cdc25 expression was insufficient to drive mitotic entry in the setting of limiting cyclin A [19], 'knockdown' of Wee1 was, indicating that Wee1-mediated Cdk1 phosphorylation is rate limiting when cyclin A function is compromised. Finally, targeted deletion of the cyclin A2 gene was observed to be lethal in the mouse embryo and the adult, associated with accumulation of some cells in G2 [20]. These observations point to a role for cyclin A/Cdk2 complexes in antagonizing Cdk1 Y15 phosphorylation and driving mitotic entry. Nonetheless, the mechanism(s) remained unclear.

### Cyclin A/Cdk2 complexes bind Wee1

We asked whether cyclin A/Cdk2 complexes might antagonize Cdk1 Y15 phosphorylation by directly binding and inactivating Wee1. Wee1 was present in cyclin A and Cdk2 immunoprecipitates from U2-OS cells and associated efficiently with exogenously expressed Cdk2 [21]. In contrast, Wee1 was less abundant in cyclin E immunoprecipitates, normalized for Cdk2 content. Cyclin E is expressed in S and G2 phase U2-OS cells, when Wee1 expression is robust. Thus, the preferential association of Wee1 with cyclin A/Cdk2 complexes over cyclin E/Cdk2 complexes suggested that the cyclin might dictate the association and that Wee1 might be recognized as a cyclin A/Cdk2 substrate.

### Conserved Cyclin A binding motifs in Wee1

Cyclin A/Cdk2 complexes are known to preferentially recognize some substrates via short sequence motifs termed 'Cy' or RxL' motifs [22-25]. Human somatic Wee1 contains four RxL sequences that are conserved throughout vertebrate somatic Wee1 proteins. Two are within the kinase domain and may contribute to kinase activity. One is in the short carboxy-terminus and one in the NRD. The latter, RxL1, is the most conserved. It is present in Drosophila Wee1 and is followed by a hydrophobic residue in the +5 position, a favored feature for cyclin A binding. The NRD has not been crystallized, and its structure is unknown. Indeed, calculations of potential order, based on primary sequence content, suggest that the NRD is generally disordered [21]. The region surrounding RxL1, however, stood out for its potential order. This circumstantial evidence suggested that RxL1 might serve a conserved functional role in Wee1. Consistent with this notion, another prominent region of predicted order in the NRD was nearby and contained the Wee box and T239. Based on evidence from studies in Xenopus, a role of RxL1 might be to direct phosphorylation and inactivation of the Wee1 box.

### Role for RxL1 in cyclin A/Cdk2 binding and phosphorylation of Wee1

We mutated each of the RxL sequences in Wee1, singly and in combination, and examined the impact on cyclin A/Cdk2 binding and T239 phosphorylation. Mutations of the RxL sequences diminished stable association of Wee1 with cyclin A/Cdk2 complexes in vivo and in vitro [21]. Mutation of RxL1 preferentially reduced T239 phosphorylation. Consistent with loss of inhibitory regulation, expression of the RxL1 and T239 mutants, respectively, resulted in greater phosphorylation of Cdk1 Y15 than wild type Wee1. Moreover, transient transfection of RxL1 and T239A mutants was each associated with an increased fraction of cells in G2 phase. These observations suggest that cyclin A/Cdk2 complexes inhibit Wee1 activity via binding to RxL1 and phosphorylation of T239 and define a molecular pathway through which cyclin A/Cdk2 complexes drive mitotic entry. Consistent with these *in vivo *data, studies in human somatic cell extracts showed that recombinant cyclin A can direct the phosphorylation and inactivation of Wee1 more efficiently than cyclin B, and combined addition of cyclins A and B induced nuclear envelope breakdown more efficiently than addition of either cyclin alone [26].

### Increased nuclear localization of RxL1 mutant

The RxL1 mutant appeared to be modestly more potent than the T239A mutant in mediating G2 phase arrest under the conditions examined, suggesting that loss of the RxL1 site might impact more than phosphorylation of T239. We therefore examined other properties of Wee1. Wee1 has been described as being localized to the nucleus during interphase and the cytoplasm during mitosis. Whether this redistribution is a cause or effect of nuclear envelope breakdown has remained unclear. Early studies demonstrated that nuclear Wee1 could potently protect cells from premature mitosis, even in the presence of activated cyclin B/Cdk1 complexes in the cytoplasm [27]. These studies implied that the nucleus was an important site of action of Wee1. Examination of their subcellular localization showed that transfected Wee1 wt and T239A exhibited a range of locations, from the nucleus to the cytoplasm [21]. In striking contrast, the RxL1 mutant was almost exclusively nuclear.

### RxL1 is embedded within a nuclear export signal

**Figure 3:**
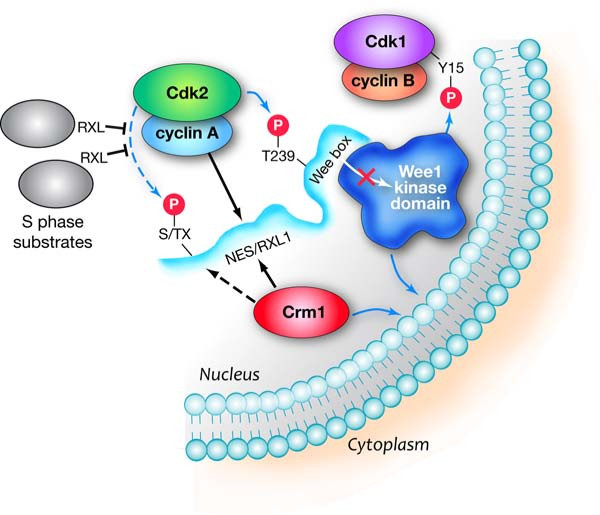
**Model for regulation of Wee1 by cyclin A/Cdk2 complexes**. Wee1 is depicted as a cyclin B/Cdk1 Y15 kinase with a globular kinase domain (dark blue) and a relatively unstructured NRD (light blue). The Cyclin A/Cdk2 complex binds RxL1 and phosphorylates T239. It might also phosphorylate a Wee1 residue that contributes to Crm1 binding (dashed arrow to S/TX). Crm1 binds the NES and possibly the additional cyclin A/Cdk2 phosphorylation site (dashed line) and mediates Wee1 export from the nucleus. S phase substrates possessing RxL motifs compete with cyclin A/Cdk2 complexes for binding to Wee1.

The simplest explanation for the restricted nuclear localization of the RxL1 mutant was that this site directs cyclin A/Cdk2-mediated phosphorylation of another residue(s) that drives Wee1 cytoplasmic redistribution. This scenario may yet prove to be true, but examination of the Wee1 primary structure suggested another potential explanation. The RxL1 sequence overlaps with residues that match the loose consensus Crm1-dependent nuclear export signal (NES) [28-30]. Subsequent experiments supported the notion that Wee1 undergoes Crm1-dependent nuclear export. Wee1 and Crm1 co-immunoprecipitated, pointing to their physical association. Leptomycin B, a Crm1 inhibitor, blocked Wee1 export. Finally, independent mutation of candidate NES sequences amino-terminal of RxL1 (but still within the small conserved, potentially structured region) markedly reduced Crm1 association and Wee1 export. In contrast, T239 phosphorylation was unaffected. These results suggest that the RxL1 region also serves as a Crm1 binding site, directing export of Wee1 from the nucleus. In addition, the decreased Wee1 T239 phosphorylation seen in an RxL1 mutant is not a secondary effect of increased nuclear retention. On the other hand, RxL1 and cyclin A/Cdk2 complexes may play a role in Wee1 export, because inhibiting these Cdk2 complexes by different means augmented Wee1 nuclear localization [21]. Previous studies suggest that phosphorylation of a carboxy-terminal Wee1 residue, S642, fosters 14-3-3 binding and cytoplasmic localization [31]. This pathway may act in parallel to the Cdk2/NES/Crm1 pathway. Most of the experiments on S642 were carried out using a Wee1 mutant lacking the Crm1-dependent NES, so the function of S642 phosphorylation in the context of the intact protein requires further definition. In summary, these results provide evidence for a bifunctional region encompassing RxL1 and the NES that mediates binding of cyclin A/Cdk2 complexes and Crm1, respectively. These interactions result in Wee box phosphorylation, inhibition of kinase activity, and nuclear export of somatic Wee1 (Fig 3).

## Discussion: New insights into Wee1 regulation

One can draw a number of conclusions from this recent work. First, redistribution of human somatic Wee1 to the cytoplasm is an active, temporally regulated event, rather than a passive byproduct of nuclear envelope breakdown. Based on conservation of NES consensus sequences among vertebrate somatic Wee1 proteins, nuclear export might also be conserved, suggesting the presence of selection pressure to maintain it. Given the lack of conservation of the NES in embryonic proteins, regulated export of Wee1 may exert an additional constraint on mitotic entry unique to somatic cells. In this light, it was somewhat surprising that expression of the NES mutant did not impose increased G2 arrest under the conditions tested [21]. It seems probable that a functional role will be uncovered by further experimentation. Second, RxL1 directs phosphorylation of T239, a modification that inhibits Cdk1 Y15 phosphorylation by Wee1. The Xenopus studies suggest that T239 phosphorylation antagonizes the function of the Wee box. Taken together, these studies have defined a direct molecular pathway through which cyclin A/Cdk2 complexes drive mitotic entry. Third, it may not be coincidence that the RxL1 cyclin A/Cdk2 binding site resides within the Crm1 binding site. Indeed, inhibition of Cyclin A/Cdk2 activity appears to reduce Wee1 export [21]. The mechanism may be direct or indirect. For example, these Cdk2 complexes could modify components of the export pathway or inactivate a nuclear localization sequence in Wee1. However, the simplest model is that RxL1 directs an additional cyclin A/Cdk2 phosphorylation(s) in Wee1 that fosters export (dashed lines in Fig 3).

Might cyclin A/Cdk1 complexes also bind RxL1 and inactivate Wee1? Cdk2 appears generally to be the preferred binding partner for cyclin A when Cdk1 and Cdk2 are expressed at their normal levels [17,18,32]. However, Cdk1 binds a small but increasing fraction of cyclin A during S and G phases [32]. Thus, cyclin A/Cdk1 complexes may contribute to Wee1 inactivation in normal cells and likely do so in cells rendered Cdk2-deficient by 'knockdown' or deletion of the Cdk2 gene [33,34]. Indeed, Cdk1 appears to be the most potent histone H3 kinase partner for recombinant cyclin A in HeLa S3 cell extracts [26]. Similarly, cyclin A is the dominant S and G2 phase cyclin and is required for cell cycle progression in many cell types, but compensatory increases in cyclin E in some cells rendered cyclin A deficient might fill the void [20]. Cyclin E has shown binding activity toward certain RxL motifs [23,35].

## Conclusions: Reconciliation of cyclin A/Cdk2 complexes as both S and M phase drivers

The conclusion that cyclin A/Cdk2 complexes drive mitotic entry raises an apparent conundrum. These complexes also drive DNA synthesis [17,20,36], and it is critical that DNA synthesis be completed prior to mitosis. This conceptual stumbling block likely accounts in part for the prior exclusion of cyclin A/Cdk2 complexes from most models of mitotic entry. The conundrum can, in principle, be solved in several ways. One model is that inactivation of Wee1 by cyclin A/Cdk2 complexes is necessary but insufficient for initiation of mitosis. In this model, cyclin A/Cdk2-mediated inactivation of Wee1 may license mitotic entry, without being the final trigger. For example, Cdc25 phosphatases may need to be activated as well [18,26]. An alternative model is that Wee1 inactivation is 'ultrasensitive' to cyclin A/Cdk2 activity. In this case, inactivation of Wee1 may require accumulation of cyclin A/Cdk2 activity beyond a threshold. As part of this model, Wee1 phosphorylation at multiple sites may be needed to effect a switch-like inactivation of the kinase [37,38]. This model is similar to the inactivation of Sic1 by Cdk1 complexes that drives S phase progression in yeast [39]. Nuclear export and/or Cyclin B/Cdk1-mediated phosphorylation might complete Wee1 inactivation in some settings. Evidence for ultrasensitivity in Xenopus embryonic Wee1 inactivation by cyclin B/Cdk1 complexes has been obtained [37,38]. Swe1 phosphorylation by Cdk1 also appears to be ultrasensitive (D. Kellogg, unpublished). Moreover, Ferrell and co-workers have developed evidence that competition among Cdk1 substrates can contribute to an ultra-sensitive response [37]. This model appears to be well suited to apply to regulation of Wee1 by cyclin A/Cdk2 complexes. Competition with the many other cyclin A/Cdk2 binding partners during DNA synthesis might help prevent premature inactivation of Wee1 and restrain mitotic entry until the time is propitious (Fig 3). Other pathways, downstream of or parallel to Wee1, almost certainly contribute to mitotic entry. In addition, other kinases may contribute to Wee1 NRD phosphorylation [40]. Given the significance of the decision to enter mitosis, the diversity of relevant inputs, and the flexibility of protein based regulatory systems [41,42]; it would seem unwise to underestimate the sophistication of the choreography.

## Future directions

There are a number of issues that merit further study. The bifunctional nature of the NES/RxL1 site raises the question of whether physical occupancy of the site by cyclin A/Cdk2 complexes competes with that of Crm1. The stable association of Wee1 with both binding partners [21] suggests this potential. In budding yeast, Cdk1 complexes appear to eventually drive their own dissociation from Wee1, by phosphorylation of the latter [1]. Therefore, high-level Cdk activity might shift the balance of such competition. Another issue is whether Wee1 can phosphorylate Cdk2 on Y15 in stable complexes with cyclin A/Cdk2. Substantial evidence has accrued that Cdk2 undergoes some Y15 phosphorylation and that this modification can be limiting for Cdk2 activity, particularly after DNA damage [43-48]. Swe1 can phosphorylate Cdk1 in stable complexes [1]. However, the Swe1 NRD is relatively divergent from the NRDs of higher eukaryotes, and cyclin A/Cdk2 complexes might bind Wee1 in an orientation that is not permissive for Cdk2 Y15 phosphorylation. Another issue is whether cyclin A/Cdk2 complexes might activate Wee1 by phosphorylation. The Swe1 response to phosphorylation by Cdk1 in budding yeast is biphasic in that Wee1 is initially activated by Clb/Cdk1 phosphorylation, with stabilization of the Swe1-Cdk complex, before being eventually inactivated and dissociated [1]. Consistent with these findings, inactive Cdk2 complexes show reduced binding to Wee1 in human cells [21]. Initial evidence for activation of human Wee1 by cyclin B/Cdk1 complexes has been obtained in somatic cell extracts [26].

## Competing interests

The authors declare that they have no competing interests.
